# Eco-Friendly Cellulose/Polyaniline Sponge for Water Remediation

**DOI:** 10.3390/ma19071381

**Published:** 2026-03-31

**Authors:** Juan C. Medina-Llamas, Fátima D. G. Rodríguez-Flores, Isaac Olvera-López, José García-Elías, María Medina-Llamas, Alicia E. Chávez-Guajardo

**Affiliations:** 1 Centro de Estudios Científicos y Tecnológicos No. 18, Instituto Politécnico Nacional, Zacatecas 98160, Mexico; 2 Unidad Académica de Ciencias de la Tierra, Universidad Autónoma de Zacatecas, Zacatecas 98058, Mexico; dariannardz3@gmail.com (F.D.G.R.-F.); isaacolvera352@gmail.com (I.O.-L.); 3 Unidad Académica de Ciencias Químicas, Universidad Autónoma de Zacatecas, Zacatecas 98058, Mexico; josegarciae@uaz.edu.mx; 4 Unidad Académica Preparatoria, Plantel II, Universidad Autónoma de Zacatecas, Zacatecas 98068, Mexico; maria.medina@uaz.edu.mx; 5 Unidad Académica de Ingeniería Eléctrica, Universidad Autónoma de Zacatecas, Zacatecas 98068, Mexico

**Keywords:** adsorption, dyes, cellulose sponge, conductive polymers, methyl orange, polyaniline

## Abstract

This work describes the fabrication of an eco-friendly sponge for the removal of dyes from aqueous solutions. For this purpose, a reused cellulose sponge (CS) that is commercially sold for makeup was covered with polyaniline (PANI), a conductive polymer that allows the addition of functional groups that are compatible with dyes present in aqueous solutions. An SEM analysis showed the successful deposition of PANI over CS fibers and confirmed that the porosity of the sponge remained after the polymerization step. The adsorption performance of the PANI-CS was evaluated in batch mode using methyl orange (MO). The adsorption capacity was 308 mg/g at pH 4.0 and after 110 min. PANI-CS achieved an adsorption percentage of 84% (*C_o_* = 25 mg/L MO) after only 20 min. The experimental data were adjusted to different isotherm adsorption models; the best fit was obtained using the Halsey model. Furthermore, the adsorption performance of PANI-CS was studied in continuous mode using a bespoke adsorption column with recirculation. The results indicated that after 5 min of interaction time, 59% of the initial MO concentration (25 mg/L) was adsorbed. These results show the potential of PANI-CS as an inexpensive adsorbent for large-scale adsorption of dyes from aqueous media.

## 1. Introduction

Industrial wastewater has a broad spectrum of pollutants, and organic dyes are among the most significant sources of industrial wastewater contamination [1]. Dyes are used in the paint, textile, leather, and cosmetic industries. The textile industry generates large quantities of dyes that are released into wastewater due to the low absorbance of dyes by the textiles [2]. Actually, more than 100,000 dyes have been synthesized, with an estimated production of 70 × 10^5^ tons/year [3]. The USA-EPA reports that a textile mill that produces ~1 ton/day of textiles consumes up to 36,000 L of water [4]. The wastewater discharged by the textile industry produces effluents characterized by high values of pollution indicators such as suspended solids, chemical oxygen demand (COD), biochemical oxygen demand (BOD), and color [5]. The USA-EPA has set a COD upper limit of 163 kg/ton of fabric per day [4]. However, reports in the literature estimate that the COD value from textile mills could be higher [6].

The presence of dyes in water is highly visible even when the dye concentration is lower than 1 ppm. In addition, their presence in water can be harmful to humans and aquatic life. For humans, exposure to dyes causes dysfunction of the liver, kidneys, brain, nervous system, and reproductive system [7]. Currently, there are several technologies to remove dyes from wastewater; among them are membrane filtration [8], electrochemical treatment [9], photocatalysis [10], ion exchange [11], and adsorption [12]. Adsorption is an effective technology with low operating costs [13]. In the literature, there is a variety of materials for dye adsorption from water; among them are inorganic materials (TiO_2_, g-C_3_N_4_, Fe_3_O_4_) [14,15], carbon-based materials (graphene and CNTs) [16,17,18], and conductive polymers (poli(3,4-etilendioxitiofeno) (PEDOT), polyaniline (PANI), polypyrrole (PPY)) [1,19,20]. Although the latter materials can be used in slurry reactors, their implementation has some drawbacks, such as high energy consumption from stirring, agglomeration of nanomaterials, and the installation and operation of additional equipment to separate the nanoadsorbents from the treated water. These factors hinder the use of suspended nanoadsorbents for industrial applications. Nevertheless, their immobilization on a support could address these latter drawbacks. In addition, the use of supported adsorbents will allow a continuous operation mode and a reduction in operation costs [21].

Recently, conducting polymers (CPs) have caught attention for a wide range of applications such as sensors [22,23], photocatalysis [24], solar cells [25], DNA purification [26], drug release materials [27], and adsorption of contaminants [15]. CPs have a conductive main chain structure due to the π-conjugation system along their backbone, which allows charge transport when charge carriers are introduced. Furthermore, the doping process in CPs occurs through oxidation (p-type doping) or reduction (n-type doping), where electrons are removed from or added to the polymer chain, respectively. This process increases the number of mobile charge carriers and enables charge delocalization, significantly enhancing electrical conductivity and modifying the chemical properties of the material [28,29]. Although the use of CPs for dye adsorption has been previously reported in the literature, we would like to explore the immobilization of CPs on a biodegradable and inexpensive support material. Cellulose is the most abundant biopolymer on Earth, with an estimated annual production of ∼1011 tons via photosynthesis [30]; it is a versatile, inexpensive, and widely available natural polymer that can be used in wastewater treatment technology due to its chemical properties. Specifically, cellulose sponges can be considered an attractive support due to their high porosity, low density, and large surface area. Although there are several reports in the literature that use CPs for the adsorption of dyes in synthetic water, the immobilization of these polymers over a natural sponge for the adsorption of dyes has been scarcely reported.

This work describes the synthesis and characterization of a polyaniline-coated cellulose sponge (PANI-CS) for the adsorption of methyl orange (MO) from aqueous media. The experiments were carried out in both batch and continuous setups with a recirculation to show the potential of PANI-CS in a continuous adsorption reactor. The obtained results demonstrate the potential of PANI-CS as an inexpensive adsorbent for large-scale adsorption of dyes. It is important to note that the immobilization of conductive polymers on a CS for dye adsorption is not commonly reported.

## 2. Materials and Methods

### 2.1. Materials

Cellulose sponge is commercially available from Patelai (New Paris, IN, USA). Aniline (99%) and sulfuric acid (H_2_SO_4_, 95%) were purchased from Sigma-Aldrich (St. Louis, MO, USA). Ammonium persulfate (APS, 98%) was purchased from J.T. Baker (Phillipsburg, NJ, USA). Methyl orange (MO) was purchased from Hycel (Zapopan, Mexico). All reagents used in this work were of analytical grade and used as received. However, aniline was distilled prior to use. Deionized (DI) water was used for the preparation of all the solutions.

### 2.2. Synthesis of Polyaniline-Coated Cellulose Sponge (PANI-CS)

**Figure 1:**
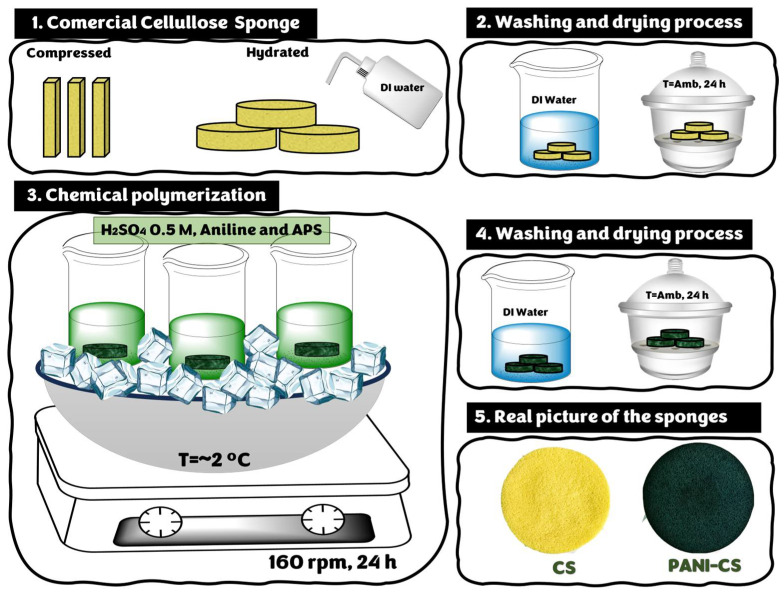
Scheme of the synthesis procedure of the PANI-CS.

First, the CS was hydrated by adding DI water and then washed using plenty of DI water to remove impurities. Subsequently, CS was dried at room temperature for 24 h. For the in situ polymerization of aniline, CS was placed in a 500 mL glass beaker containing 150 μL aniline and 100 mL of 0.5 M H_2_SO_4_. The mixture was kept under agitation using an orbital shaker (Scientific CVP-2000P, Thermo Fisher Scientific, Sterling, VA, USA) at 150 rpm and at low temperature (T~2 °C). After 10 min, a solution containing 20 mL of 0.5 M H_2_SO_4_ and 0.75 g of APS was added. The reaction proceeded for 24 h under agitation and low temperature. The PANI-CS was retrieved and washed three times with DI water and subsequently dried at room temperature for 24 h. Figure 1 illustrates a scheme of the synthesis procedure.

### 2.3. Adsorption Experiments

**Figure 2:**
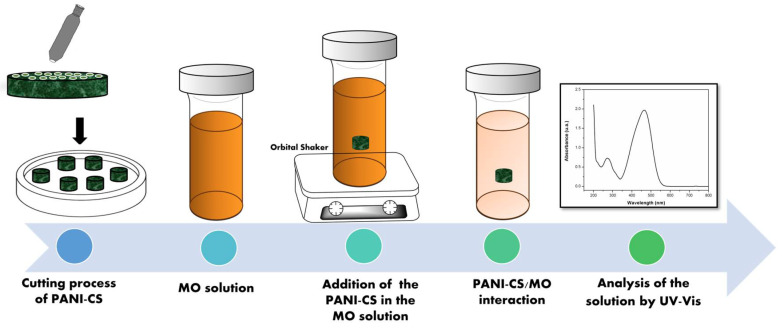
Scheme of the MO adsorption process by the PANI-CS.

The adsorption capacity of PANI-CS was evaluated for the removal of MO under different conditions, such as interaction time, pH, and initial concentration of MO. For each experiment, a small piece of PANI-CS was cut (0.04 g) and added to a glass vial containing 10 mL of MO solution with a known concentration (mg/L) under constant agitation (150 rpm). The concentration of the dye before and after each experiment was analyzed by UV-Vis spectrophotometry. Adsorption experiments using the pristine cellulose sponge were conducted under the same experimental conditions. The adsorption percentage of the PANI-CS was evaluated as a function of the solution pH. For this experiment, the pH of different MO solutions was adjusted from 2.0 to 10.0 by adding small aliquots of either HCl or NaOH solutions. The initial concentration of the solution was 25 mg/L MO, and the interaction time was 30 min. Additional experiments were carried out by modifying the adsorption time (0–120 min) using a 25 mg/L MO solution. The concentration of MO in the solution before and after each experiment was determined using UV-Vis spectrophotometry by obtaining the spectrum and determining the highest absorbance peak. A volume of 2 mL of each MO solution was used for each measurement. The selected wavelengths were 486 nm for solutions with pH < 3.0 and 461 nm for solutions within the pH range of 4.0–10.0. Figure 2 shows a scheme of the MO adsorption process by the PANI-CS.

The adsorption percentage was determined from the following equation:

(1) $$\%\;Adsorption=\frac{\left(C_{0}-C_{f}\right)}{C_{0}}\cdot 100,$$

where *C*_0_ is the initial concentration, and *C_f_* is the final concentration (mg/L) of methyl orange. The adsorption capacity (*q_e_*) of PANI-CS was obtained using the following equation:

(2) $$q_{e}=\frac{\left(C_{0}-C_{e}\right)V}{m},$$

where *q_e_* is the adsorption capacity (mg_dye_/g_adsorbent_), *m* is the mass of adsorbent (g), and *V* is the volume of the solution (L). The adsorption capacity as a function of time (*q_t_*) can be calculated from the following equation:

(3) $$q_{t}=\frac{\left(C_{0}-C_{t}\right)V}{m},$$

where *C_t_* is the concentration of dye (MO) in a specific adsorption time (min).

### 2.4. Adsorption and Desorption Experiments

The adsorption process was performed by placing a PANI-CS in a beaker with 10 mL of 25 mg/L MO, under agitation for 1 h at 150 rpm. Next, the PANI-CS was removed from the solution, and the dye concentration was determined by UV-Vis spectrophotometry. From this experiment, the concentration of MO adsorbed on the PANI-CS was calculated. To start the desorption process, the PANI-CS previously used in the adsorption experiment was immersed in 20 mL of 1.0 M NaOH to promote the desorption of MO. The interaction time for the desorption step was 1 h at 150 rpm. Finally, PANI-CS was removed from the solution, and the dye concentration was analyzed. For this work, four consecutive cycles of adsorption/desorption were performed using the same piece of PANI-CS.

### 2.5. Continuous Adsorption Experiments

The adsorption performance of PANI-CS was evaluated in a continuous mode using a bespoke adsorption column with recirculation. Figure S1a shows a picture of the adsorption column. For the experiment, 200 mL of MO solution (25 mg/L) at pH = 4 was recirculated at a constant flow using a small water pump, and 0.3 g of PANI-CS was loaded in the metal fitting used to place the sponge (Figure S1b). The adsorption process was monitored by taking 1.0 mL aliquots at specific time intervals. The dye concentration in the solution was determined at λ = 501 nm using a UV-Vis spectrophotometer.

### 2.6. Characterization Methods

The CS and PANI-CS were characterized using FTIR spectroscopy and SEM. The Attenuated Total Reflectance–Fourier Transform Infrared (ATR-FTIR) spectra of the samples were recorded from 4000 cm^−1^ to 600 cm^−1^ using an IFS-66 Bruker (Madison, WI, USA) spectrophotometer. The SEM micrographs were acquired using a VEGA 3 TESCAN (Fuveau, France). For the SEM sample preparation, a piece of sponge (pristine CS or PANI-CS) measuring approximately 0.25 cm^2^ was placed on a carbon tape attached to an SEM sample holder. A gold film approximately 10 nm thick was deposited using a Cressington 108 Series Sputter Coater (Cressington Scientific Instruments, Watford, UK). The MO concentration in the solution was estimated by using a UV-Vis Perkin Elmer XLS (Shelton, CT, USA). The wavelength used to estimate the concentration of MO was the highest absorbance peak from the MO UV-Vis spectrum.

## 3. Results and Discussion

### 3.1. Synthesis of PANI-CS

**Figure 3:**
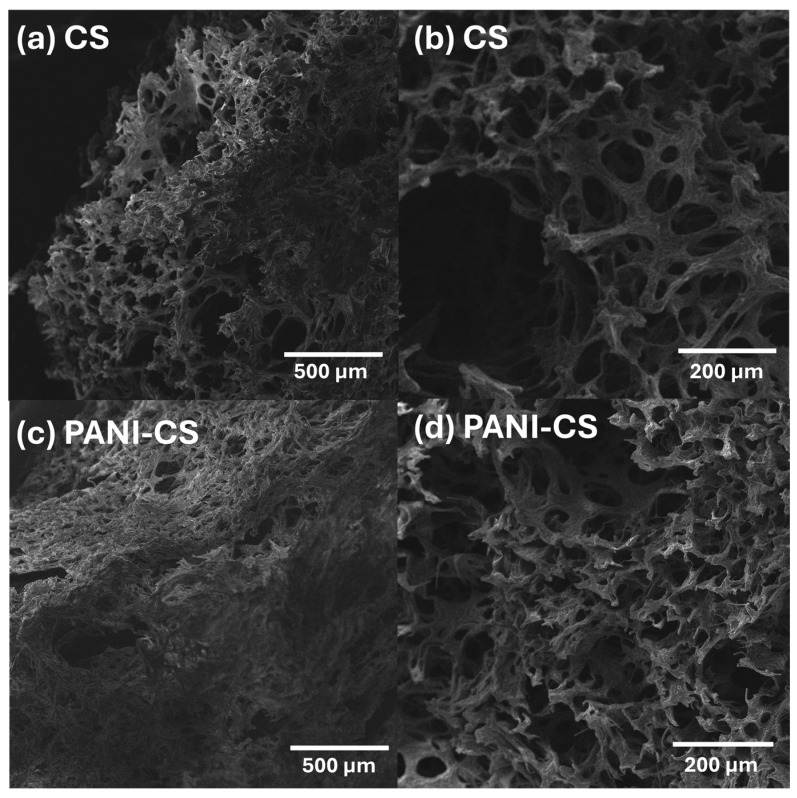
SEM micrographs of the pristine CS (**a**,**b**) and PANI-CS (**c**,**d**).

As mentioned in the Section 2, a commercial cellulose sponge was coated with PANI by the in situ polymerization of aniline in acidic media. The SEM micrographs in Figure 3a,b show the morphology of the pristine CS, while Figure 3c,d shows the morphology of the PANI-CS at different magnifications to highlight that the porosity of the material is still retained after the polymerization of aniline. Overall, the micrographs show the extremely porous structure of CS and how its porosity is slightly reduced by the polyaniline coating over the cellulose fibers. Nevertheless, a highly porous structure remains, which will allow the mass transfer of MO from the aqueous solution to the surface of PANI-CS.

**Figure 4:**
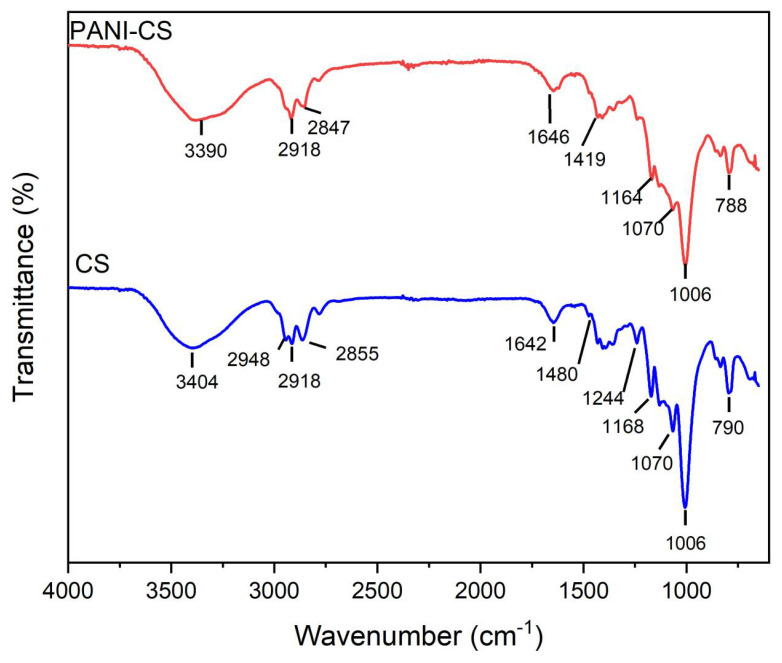
ATR-FTIR spectra of CS and PANI-CS.

To further corroborate the coating of PANI over CS, an ATR-FTIR analysis was performed for the pristine CS and the PANI-CS; the results can be found in Figure 4. First, the pristine CS (Figure 4, blue spectrum) has a broad band at 3390 cm^−1^ attributed to the O-H stretching. The peaks at 2918 and 2847 cm^−1^ are assigned to the stretching of the C-H group of hydrocarbon constituents in polysaccharides. The band at 1646 cm^−1^ corresponds to the vibration of water molecules absorbed in the cellulose sponge. The bands at 1419, 1236, 1164, and 1070 cm^−1^ belong to the bending and stretching vibrations of -CH_2_, -CH, and C-O bonds in cellulose. The band at 1006 cm^−1^ can be attributed to the C-O group of ethers and secondary alcohols from the cellulose backbone [30,31]. The peak at 788 cm^−1^ is assigned to the deformation vibrations of the C-H group in the glucose units. In Figure 4 (red spectrum), it is possible to observe the characteristic peaks of both CS and PANI, which corroborates the deposition of PANI into the sponge. The peak at 3404 cm^−1^ is wider and with a higher intensity, which is assigned to O-H stretching groups of cellulose and N-H stretching vibration of PANI. The band at 1244 cm^−1^ is related to the C-N stretching vibration of the benzenoid ring in PANI. The characteristic bands of PANI at 1435 and 1480 cm^−1^ are due to C=C stretching of the benzenoid and quinoid rings, respectively. The peaks at 2775 and 2948 cm^−1^ are due to the asymmetric and symmetric stretching of -CH_2_- groups, respectively [32].

### 3.2. Adsorption Experiments

#### 3.2.1. pH, Time, and Concentration Effect

**Figure 5:**
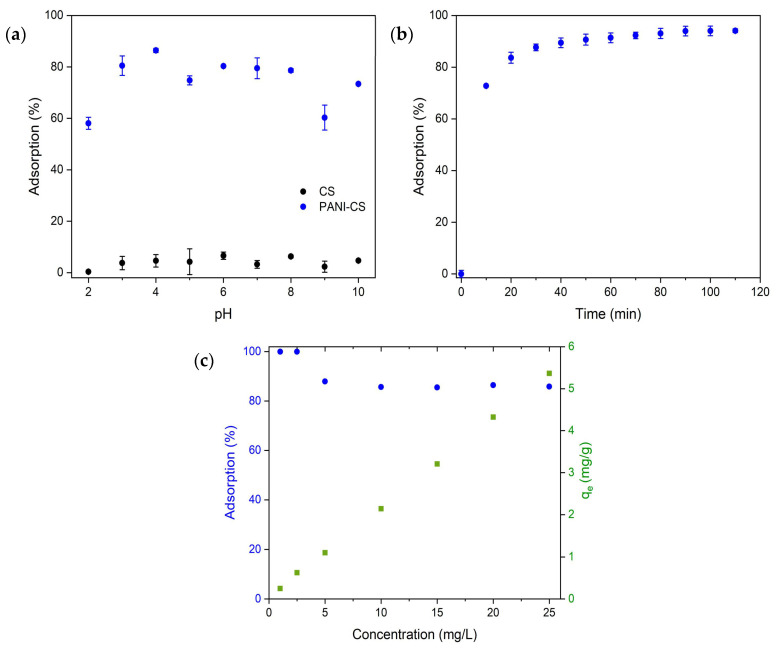
Adsorption experiments of MO using CS and the PANI-CS varying (**a**) pH, (**b**) adsorption time, and (**c**) initial MO concentration.

The adsorption capacity was evaluated as a function of the solution pH, since pH is a parameter that highly contributes to the adsorption process. For these experiments, the effect of pH on the pristine CS and on the PANI-CS was evaluated (Figure 5a). The results indicate that CS has a poor adsorption performance across all pH values. The adsorption removal of CS ranges from 0.4% at pH = 2 up to 6.6% at pH = 6. However, higher adsorption performance is observed for PANI-CS across all pH values, with a maximum adsorption percentage of 86% at pH = 4. These results indicate that PANI is the main material for dye adsorption, and the contribution of cellulose is insignificant. Therefore, pH = 4 was selected as the optimum value for the adsorption of MO. The tabulated data can be found in Table S1. Figure 5b shows the adsorption performance of the PANI-CS as a function of interaction time; the tabulated data can be found in Table S2. The results indicate that, after 10 min of interaction time, the PANI-CS can remove up to 74% of the initial MO concentration. Higher interaction time increases the percentage removal up to 91% and 97% after 1 and 2 h of interaction time, respectively. Figure 5c shows the adsorption percentage and the adsorption capacity (*q_e_*) as a function of MO concentration in the solution. The plot shows a complete removal of the dye achieved when low initial concentrations were used (1.0 and 2.5 mg/L MO). At higher initial concentrations (5.0 up to 25 mg/L), the adsorption percentages varied from 87% up to 85%, respectively. The former result is because there is an increment in the mass transfer coefficient that occurs when the concentration of dye increases.

#### 3.2.2. Adsorption Isotherms

To study the adsorption behavior of the PANI-CS, the experimental adsorption data were fitted to the Langmuir, Freundlich, Temkin, and Halsey models. The Langmuir model in its linear form can be described as

(4) $$\frac{C_{e}}{q_{e}}=\frac{1}{{bq}_{m}}+\frac{C_{e}}{q_{m}},$$

where *q_e_* represents the amount of MO adsorbed per gram of PANI-CS (mg/g), *C_e_* is the MO adsorption equilibrium concentration (mg/L), *q_m_* is the maximum adsorption capacity (mg/g), and *b* is the Langmuir constant (L/mg). The Langmuir model proposes a monolayer adsorption mechanism, with a finite number of adsorption sites in a homogeneous surface [33].

The Freundlich isotherm model in its linearized form can be written as

(5) $$\log q_{e}=\log K_{F}+\frac{1}{n}\log C_{e},$$

where *K_F_* is a constant related to the adsorption capacity of PANI-CS and *n* is a constant related to the intensity of the adsorption. The former model proposes a multilayer adsorption process with a heterogeneous distribution of the active sites [34].

The linearized Temkin isotherm model can be written as

(6) $$q_{e}=\mathrm{Bln}k_{T}+B\ln C_{e},$$

where *k_T_* y *B* are constants related to the maximum binding energy and heat of adsorption, respectively. This model considers the indirect effects of adsorbate [35].

The linearized Halsey equation can be written as

(7) lnqe=1nlnβ+1n ln lnCsCe       β=kqmn,

where *k* is a constant related to the adsorption capacity of the PANI-CS and y *C_e_* is the solute solubility.

**Figure 6:**
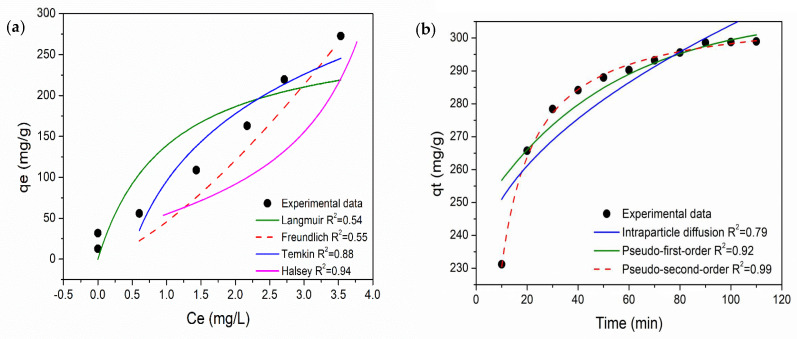
Experimental adsorption data adjusted to different (**a**) isotherm models and (**b**) kinetic models.

The fitting parameters and correlation coefficients (R^2^) obtained for each model are presented in Table 1. Among the evaluated models, the Halsey isotherm showed the best fit to the experimental data, indicating the adsorption process occurs on a heterogeneous surface and involves multilayer formation [36]. This behavior is consistent with the structural characteristics of PANI-CS, which presents a non-uniform distribution of active sites due to the polymer coating on the cellulose substrate. Although the Halsey model provided the highest correlation coefficient, a slight deviation between the experimental data and the fitted curve was observed (Figure 6a). This deviation can be attributed to the complexity of the adsorption system, where multiple mechanisms may coexist, including surface adsorption and diffusion processes. Consequently, the intrinsic heterogeneity of the PANI-CS and possible pore size distribution effects may not be fully captured by the model assumptions.

The Langmuir model, which assumes monolayer adsorption on a homogeneous surface, showed a lower fitting quality, suggesting that the adsorption process cannot be described by a single uniform energy level. Similarly, the Freundlich model provided a reasonable fit, supporting the presence of heterogeneous adsorption sites, but did not represent the experimental data as accurately as the Halsey model.

Overall, the isotherm analysis suggests that the adsorption of MO onto PANI-CS is a complex process involving multilayer adsorption on a heterogeneous surface, with contributions from different interaction mechanisms. Figure S2 shows the fitting data from each fit of each adsorption model.

#### 3.2.3. Statistical Analysis

To evaluate the goodness of fit between an adsorption isotherm model and the experimental adsorption equilibrium data, an error analysis was performed. In adsorption studies, the reliability of the fitting procedure requires the use of statistical functions that quantify the deviation between experimental values and those predicted by a theoretical model. In this work, the coefficient of determination (R^2^) and the chi-square (χ^2^) test were used to assess the fitting quality of the Langmuir, Freundlich, Temkin, and Halsey isotherm models. The chi-square statistic represents the sum of the squared differences between the experimental adsorption capacities and the values calculated from the theoretical model, normalized by the calculated values. The chi-square function was obtained using the following equation:

(8) $$\chi^{2}=\sum \left(\frac{{\left({q_{e}}_{exp}-{q_{e}}_{mod}\right)}^{2}}{{q_{e}}_{mod}}\right),$$

where ${q_{e}}_{exp}\;$is the experimentally determined adsorption capacity of the PANI-CS at equilibrium (mg/g), and ${q_{e}}_{mod}\;$is the adsorption capacity calculated by the corresponding isotherm model (mg/g) [37]. A smaller χ^2^ value indicates a closer agreement between the experimental data and the predicted values from the model, suggesting a better representation of the adsorption process. Conversely, larger χ^2^ values indicate a higher deviation between experimental and theoretical results. Therefore, the chi-square test and coefficient of determination (R^2^) were used to identify the isotherm model that best describes the adsorption behavior between MO and PANI-CS.

The obtained χ^2^ values show that the Halsey isotherm model has the lowest χ^2^ value among the evaluated models, indicating this model provides the best representation of the adsorption equilibrium of MO onto the PANI-CS. This result is consistent with the R^2^ obtained from the linear fittings, which also suggests the Halsey model describes the adsorption behavior more accurately. This result once again confirms that the adsorption process occurs on a heterogeneous surface and involves multilayer adsorption interactions between the MO molecules and the active sites of PANI-CS.

#### 3.2.4. Adsorption Kinetics

Adsorption kinetics is fundamental to understanding how the MO molecules adhere to the PANI-CS surface, and it helps to predict the capacity of PANI-CS to remove MO. For this, the results obtained were fitted to different kinetics models:

(9) $$\mathrm{Pseudo}-\mathrm{first}\;\mathrm{order}\;(\mathrm{PFO})\;\;\;\;\;\;\;\;\;\;\;\;\;\;\;\log\left(q_{e}-q_{t}\right)=\log q_{e}-\frac{k_{1}}{2.303}t\;$$

(10) $$\mathrm{Pseudo}-\sec\mathrm{ond}\;\mathrm{order}\;(\mathrm{PSO})\;\;\;\;\;\;\;\;\;\;\;\;\;\frac{t}{q_{t}}=\frac{1}{h}+\frac{1}{q_{e}}t,\;\;\;\;\;\;\;\;\;\;\;\;\;\;\;\;\;\;h=k_{2}q_{e}^{2}$$

(11) $$\mathrm{Intraparticle}\;\mathrm{diffusion}\;(\mathrm{ID}){\;\;\;\;\;\;\;\;\;\;\;\;\;\;\;\;\;\;\;\;\;\;\;q}_{t}=k_{id}{\left(t\right)}^{0.5}+C$$

where $q_{e}(mg/g)$ is the amount of MO adsorbed per gram of PANI-CS, $q_{t}(mg/g)$ is the quantity of MO adsorbed per gram of adsorbent at the equilibrium at time *t*, $k_{1}$ (min^−1^) is the adsorption rate constant, while $k_{2}$ (g/mg·min) is the corresponding adsorption rate constant, $h=k_{2}q_{e}^{2}{\cdot k}_{i}$(min^−1^) is the adsorption rate, and C is the intraparticle diffusion [38]. Table 2 shows the adsorption kinetic parameters calculated from PFO, PSO, and ID models and their correlation coefficient (R^2^) values. The PSO model had the best fit for the MO adsorption since an R^2^ value of 0.99 was obtained, while values of 0.92 and 0.79 were obtained with the other models for PFO and ID, respectively. Also, it can be highlighted that the calculated value for $q_{e}$ using this model is similar to the experimental value. Therefore, the results suggest that a chemisorption process is the adsorption mechanism between MO molecules and PANI-CS. Figure 6b shows the plot of the experimental adsorption data ($q_{t}$ versus time) and the fitting obtained using the PSO, PFO, and ID kinetic models. Figure S3 shows the graph of each linear fit to the experimental data for the three different kinetic models.

#### 3.2.5. Adsorption Mechanism

**Figure 7:**
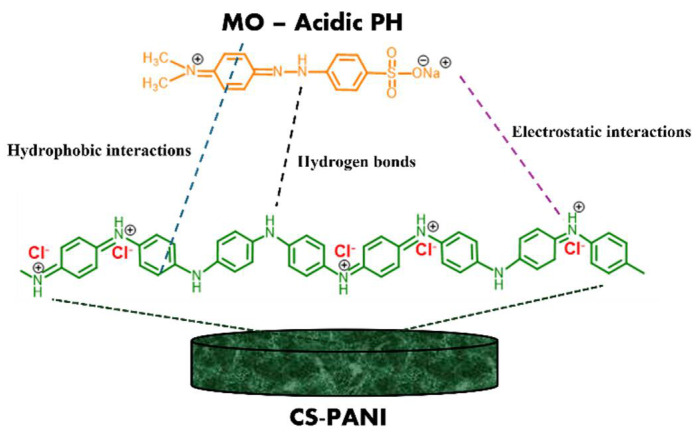
Scheme of the adsorption mechanisms between MO molecules and PANI-CS.

The adsorption mechanism behavior of MO molecules on the PANI-CS can be interpreted by considering different interaction mechanisms, including electrostatic attraction, hydrophobic interaction, and hydrogen bonding [32,34]. For instance, under acidic conditions, the negatively charged sulfonate groups of MO are able to interact with the positively charged amine sites of PANI (electrostatic interactions). In addition, the hydrogen bonds between the hydrazone groups of MO and the secondary amine of PANI. An additional mechanism is the hydrophobic interaction between the aromatic structures of MO molecules and the aromatic backbone of PANI, driven by the aggregation tendency of nonpolar moieties in aqueous media (Figure 7). The proposed adsorption mechanism is in good agreement with the kinetic and isotherm results obtained for PANI-CS. The kinetic results showed that the adsorption process is best described by the PSO model (R^2^ = 0.99), indicating that chemisorption is the dominant mechanism governing the interaction between MO molecules and the adsorbent surface. This supports the involvement of specific interactions, such as electrostatic attraction and hydrogen bonding, between the functional groups of PANI and MO. Furthermore, the adsorption isotherm data were best fitted by the Halsey model, which suggests a multilayer adsorption process occurring on a heterogeneous surface. This is consistent with the structural characteristics of PANI-CS, where a distribution of active sites is available due to the porous cellulose support and the irregular coating of PANI. Therefore, the proposed mechanism based on electrostatic interactions between protonated amine groups of PANI and the anionic sulfonate groups of MO, hydrogen bonding, and hydrophobic (π–π) interactions between aromatic structures is consistent with both the chemisorption nature indicated by the PSO model and the multilayer adsorption behavior described by the Halsey isotherm. These combined results confirm that the adsorption process involves strong surface interactions along with heterogeneous and multilayer adsorption phenomena.

#### 3.2.6. Continuous Adsorption Reactor with Recirculation

**Figure 8:**
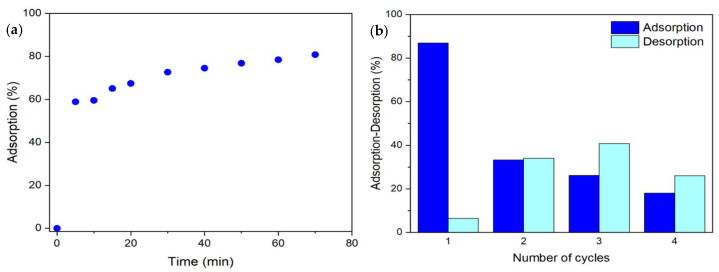
(**a**) Adsorption experiment of MO in PANI-CS using a continuous adsorption reactor with recirculation and (**b**) adsorption and desorption cycles. For the adsorption step: 10 mL at 25 mg/L MO at pH = 4. For the desorption step, 20 mL of NaOH at 1.0 M. For both experiments, the adsorption time was one hour.

The adsorption performance of PANI-CS was determined in a continuous mode using a bespoke adsorption column with recirculation. This experiment was done at pH = 4 and an initial MO concentration of 25 mg/L. Figure S1 shows a picture of the recirculation system. Figure 8a shows the adsorption performance against time, while the tabulated data can be found in Table S3. The results indicate that after 5 min of interaction time, 59% of the initial MO concentration (25 mg/L) was removed. This is a consequence of the high quantity of available active sites over the PANI-CS surface. As the experiment continued, the adsorption velocity decreased because the number of available sites decreased and the system tended to reach an adsorption equilibrium. For instance, after 1 h of interaction time, the adsorption percentage increased up to 81%.

#### 3.2.7. Reusability

A set of adsorption and desorption cycles was conducted to determine how many times the same PANI-CS can be used as an effective material for the adsorption of MO. To perform this experiment, the same PANI-CS was subjected to four adsorption–desorption cycles. A detailed description of the experiment can be found in Section 2.4, and the results are reported in Figure 8b. During the first adsorption cycle, PANI-CS was immersed in an MO solution (25 mg/L) for 1 h, reaching its maximum adsorption capacity, corresponding to 86.9% removal. Subsequently, the same piece of PANI-CS was subjected to the first desorption cycle, using a 1.0 M NaOH solution; the sample released only 6.5% of the MO molecules adsorbed at the surface from the first adsorption cycle. In the second adsorption cycle, the same PANI-CS was immersed in a fresh 25 mg/L MO solution and achieved only 33% removal. This significant decrease in adsorption performance is attributed to the incomplete desorption of MO molecules from the first desorption cycle, which left a portion of the active sites occupied and unavailable for further adsorption. During the second desorption process, 34% of MO molecules were released. Notably, this desorption percentage is higher than the % of adsorption achieved in the second cycle. This apparent increase can be explained by the release of MO molecules retained from both the first and second adsorption cycles. Consequently, the cumulative release of previously adsorbed molecules leads to a higher desorption percentage in subsequent cycles.

### 3.3. Comparison Among MO Adsorbent Materials

Table 3 reports the adsorption capacity of a wide variety of materials from the literature that have been used for MO adsorption in aqueous media and compares them to the adsorption capacity value of PANI-CS. In addition, at the bottom of Table 3, there are a couple of works from the literature that have PANI in their composition and were used to absorb MO. When analyzing Table 3, the proposed material has a higher adsorption capacity, which indicates it has great potential in the area of water treatment.

## 4. Conclusions

This work describes the use of an eco-friendly sponge for the removal of MO from aqueous media. The cellulose sponge was used as a support. Polyaniline was deposited over the CS through the in situ chemical polymerization of aniline. The composite sponge maintains its porosity after the coating stage. Batch adsorption experiments were carried out to evaluate the sponge’s ability to remove methyl orange. The PANI-CS achieved an adsorption percentage of 84% after only 20 min of interaction time. The adsorption capacity of PANI-CS was 308 mg/g. In addition, the adsorption performance of PANI-CS was evaluated in a continuous mode using a bespoke adsorption column with recirculation. The results indicate that after 5 min of interaction time, 59% of the initial MO concentration (25 mg/L) was removed. These results indicate that PANI-CS could be a low-cost adsorbent material for large-scale applications. Moreover, this gives a second use to a makeup sponge and thus reduces our footprint in the environment.

## Figures and Tables

**Table 1:** Isotherm parameters for the Langmuir, Freundlich, Temkin, and Halsey models for the MO adsorption by the PANI-CS and chi-square test $\chi^{2}$.

**Langmuir**				**Freundlich**			
***q_m_* (mg/g)**	b ** (L/mg)**	$R^{2}$	$\chi^{2}$	$K_{F}$ ** (mg/g)**	***1/n***	$R^{2}$	$\chi^{2}$
283	0.96	*0.54*	0.85	45.70	1.40	0.55	1.42
**Temkin**				**Halsey**			
$K_{T}$ ** (mg/g)**	B	$R^{2}$	$\chi^{2}$	β ** (mg/g)**	***1/n***	$R^{2}$	$\chi^{2}$
2.23	119	0.88	2.10	4 × 10^−3^	−0.90	0.94	0.39

**Table 2:** Kinetic parameters for dye (MO) adsorption onto PANI-CS.

**Pseudo-first-order (PFO)**			
*q_e,exp_* (mg/g)	*q_e,calc_* (mg/g)	$k_{1}$ (min^−1^)	$R^{2}$
308	62.5	−1.9 × 10^−2^	0.92
**Pseudo-second-order (PSO)**			
$k_{2}\;$ (g/mg min)	*q_e,calc_* (mg/g)	*H* (mg/g min)	$R^{2}$
9.6 × 10^−4^	308	91.3	0.99
**Intraparticle diffusion (ID)**			
	$K_{id}$ (min^−1^)	$R^{2}$	
	7.74	0.79	

**Table 3:** Adsorption capacity (*q_e_*) of different materials reported in the literature for the MO adsorption, and the reported value in this work by the PANI-CS.

Material (Adsorbent)	*q_e_* (mg/g)	Reference
Cationic cellulose	76.9	[39]
Cetyltrimethylammonium bromide—Ti_3_C_2_Tx MXene	213.0	[40]
Chitosan bead	12.5	[41]
MgFeAl-LTH	249.3	[42]
Zn–Co–Fe/LDH/chitosan	165.9	[43]
Egyptian doum palm	264.9	[44]
Raw clay	15.6	[45]
Commercial activated carbon	129.3	[46]
Cobalt oxide (Co_3_O_4_) cube—polyaniline	109.9	[47]
Polyurethane/polyaniline	255.0	[32]
PANI-CS	308.0	This work

## Data Availability

The original contributions presented in this study are included in the article/Supplementary Material. Further inquiries can be directed to the corresponding authors.

## Supplementary Materials

The following supporting information can be downloaded at https://www.mdpi.com/article/10.3390/ma19071381/s1. Figure S1. (a). Picture of the bespoke MO adsorption column with recirculation and (b) close-up of the fitting use to hold the PANI-CS. Table S1. Adsorption percentage of MO using the pristine CS and PANI-CS at different pH values and Co = 25 mg/L MO. Table S2. Adsorption percentage of MO using PANI-CS at different adsorption times using a *Co* = 25 mg/L MO at pH = 4. Figure S2. Linear fitting of experimental data using (a) Langmuir, (b) Freundlich, (c) Temkin, and (d) Halsey isotherm models, for MO adsorption onto PANI-CS. Figure S3. Linear fitting of experimental data using (a) pseudo-first-order, (b) pseudo-second-order, and (c) intraparticle diffusion kinetic models, for MO adsorption onto PANI-CS. Table S3. Adsorption experiment using a bespoke adsorption column with recirculation. Adsorption percentage of MO using PANI-CS against time (VTotal = 200 mL, Co = 25 mg/L MO at pH = 4).

## Abbreviations

The following abbreviations are used in this manuscript:

CS	Cellulose Sponge
PANI	Polyaniline
MO	Methyl Orange
