# Supplementary material for: Eco-Friendly Cellulose/Polyaniline Sponge for Water Remediation
Source: Materials (Basel). 2026 Mar 31;19(7):1381. doi: 10.3390/ma19071381 (PMC13074416; doi:10.3390/ma19071381)
Supplement: Supplementary file 1 [file materials-19-01381-s001.zip › materials-4191929-supplementary.pdf]

Supporting information

Eco-friendly cellulose/polyaniline sponge for water remediation

Juan C. Medina-Llamas[1]*, Fátima D. G. Rodríguez-Flores[2], Isaac Olvera-López[2], José García-Elías[3], María Medina-Llamas[4,5], Alicia E. Chávez-Guajardo[2]*

[1] Centro de Estudios Científicos y Tecnológicos No. 18, Instituto Politécnico Nacional, 98160, Zacatecas, Zac, México; jmedina@ipn.mx
[2] Unidad Académica de Ciencias de la Tierra, Universidad Autónoma de Zacatecas, 98058, Zacatecas, Zac, México; dariannardz3@gmail.com (F.D.G.R.F), isaacolvera352@gmail.com, (I.O.L), achavezg@uaz.edu.mx (A.E.C.G.)
[3] Unidad Académica de Ciencias Químicas, Universidad Autónoma de Zacatecas, 98058 Zacatecas, México; josegarciae@uaz.edu.mx
[4] Unidad Académica Preparatoria, Plantel II, Universidad Autónoma de Zacatecas, 98068, Zacatecas, Zac, México; maria.medina@uaz.edu.mx
[5] Unidad Académica de Ingeniería Eléctrica, Universidad Autónoma de Zacatecas, 98068, Zacatecas, Zac, México; maria.medina@uaz.edu.mx
* Correspondence: achavezg@uaz.edu.mx; Tel.: +52-492-922-64-70 and jmedina@ipn.mx; Tel.: +52-55- 57-29-60-00

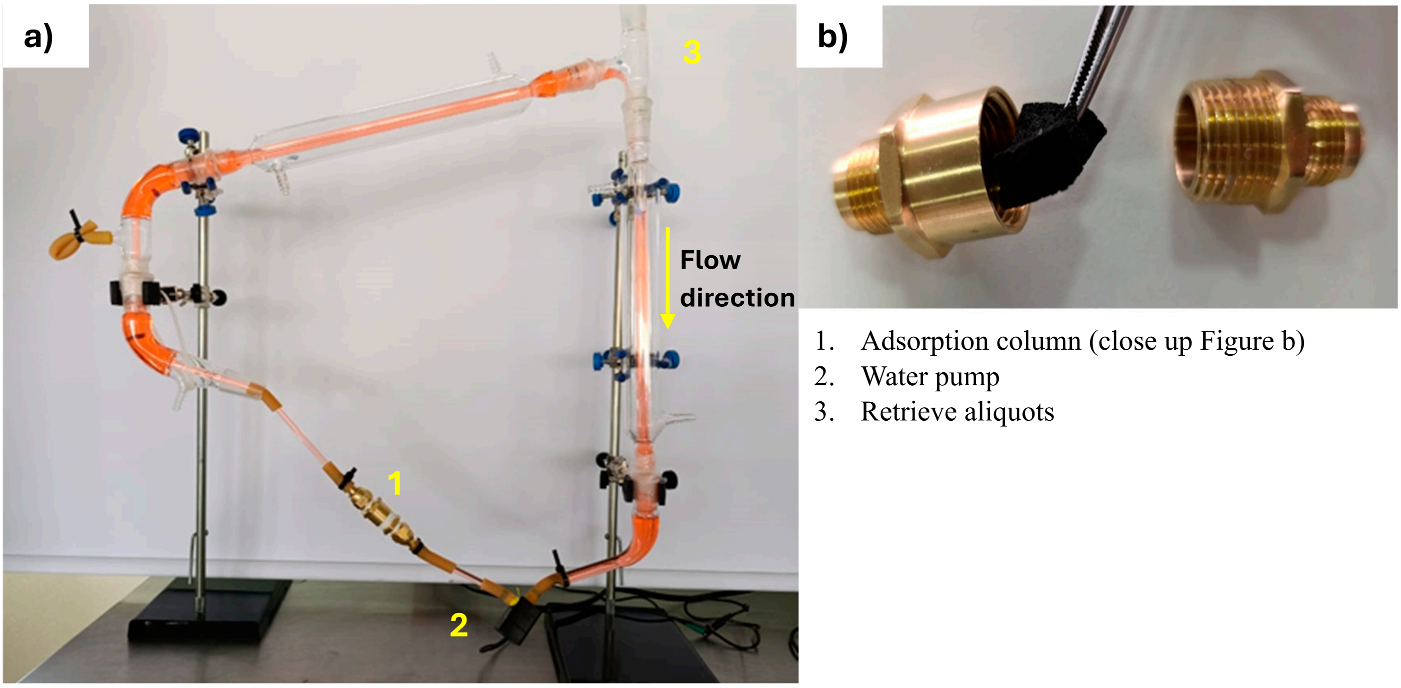

1. Adsorption column (close up Figure b)
2. Water pump
3. Retrieve aliquots

Figure S1. a) Picture of the bespoke MO adsorption column with recirculation and b) close-up of the fitting use to hold the PANI-CS.

Table S1. Adsorption percentage of MO using the pristine CS and PANI-CS at different pH values and *Co* = 25 mg/L MO.

Solution pH	% Adsorption CS	% Adsorption PANI-CS
2	0.4	58.1
3	3.8	80.5
4	4.6	86.4
5	4.2	74.7
6	6.6	80.3
7	3.2	79.5
8	6.3	78.6
9	2.3	60.3
10	4.7	73.4

Table S2. Adsorption percentage of MO using PANI-CS at different adsorption time using a *Co* = 25 mg/L MO at pH = 4.

**Time**	**% Adsorption PANI-CS**
0	0
10	72.8
20	83.6
30	87.6
40	89.4
50	90.7
60	91.4
70	92.3
80	93.0
90	94.0
100	94.1
110	94.1
120	97.0

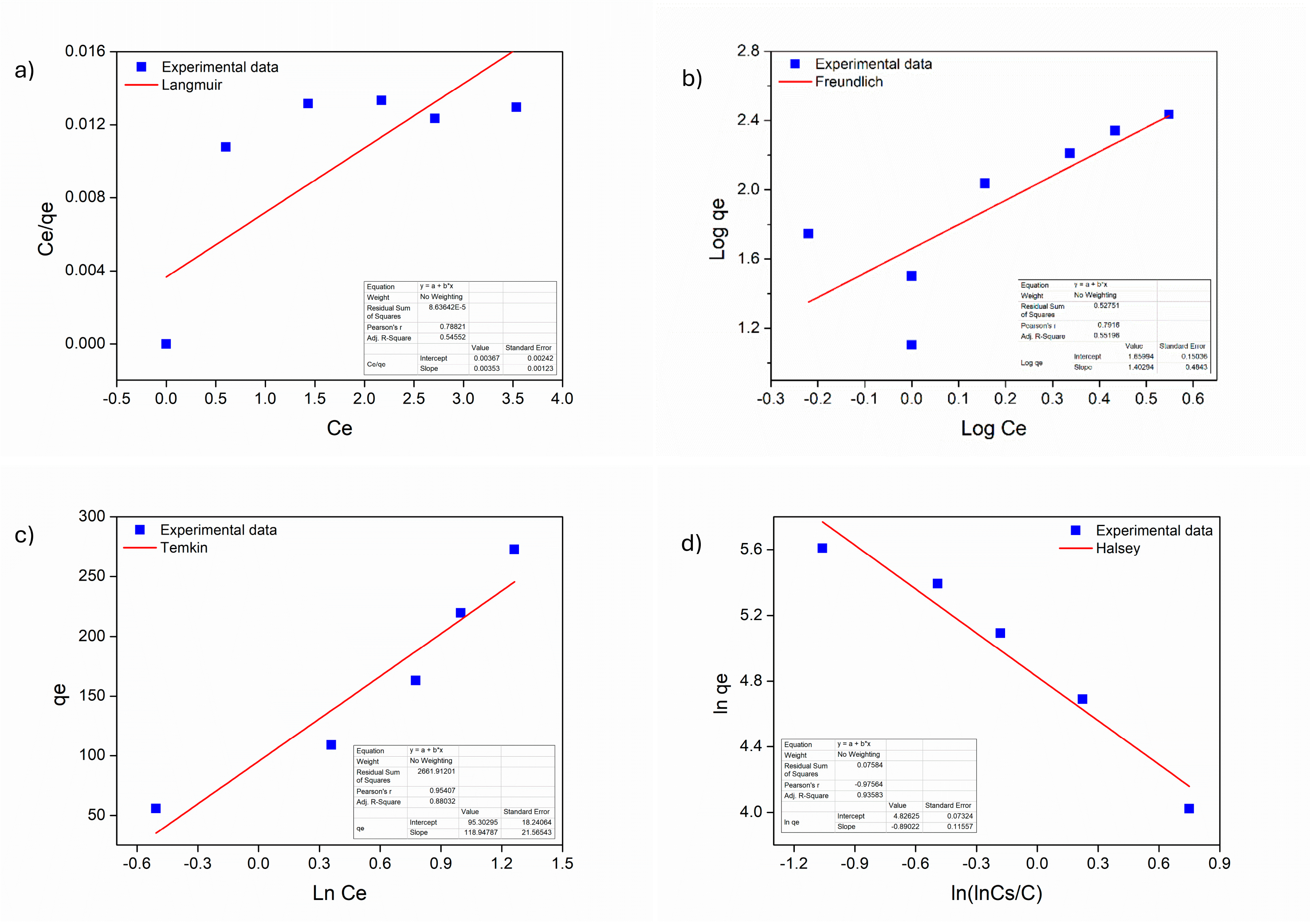

Figure S2. Linear fitting of experimental data using (a) Langmuir, (b) Freundlich, (c) Temkin and (d) Halsey isotherms models, for MO adsorption onto PANI-CS.

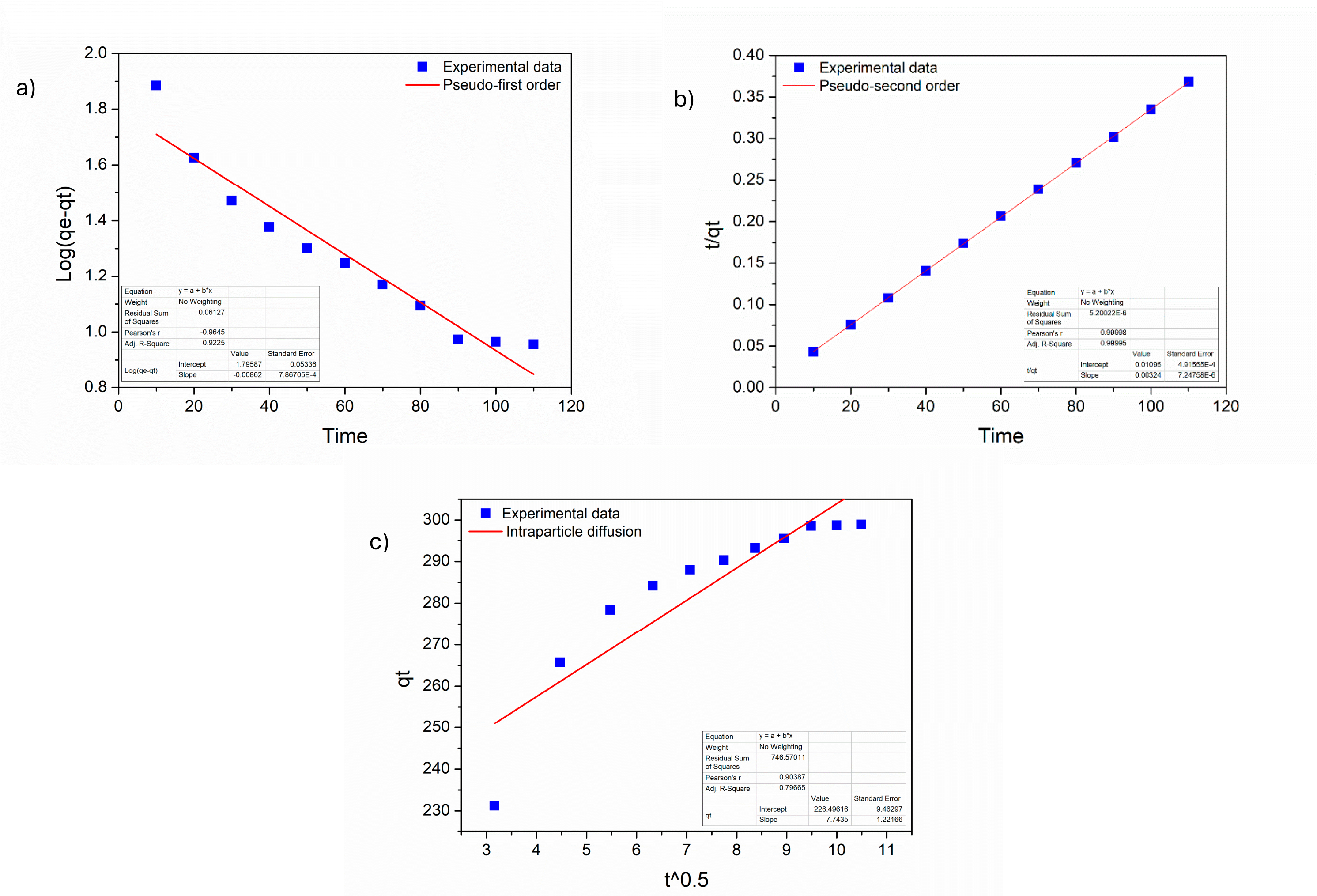

Figure S3. Linear fitting of experimental data using (a) Pseudo-first-order, (b) Pseudo-second-order, and (c) Intraparticle diffusion kinetic models, for MO adsorption onto PANI-CS.

Table S3. Adsorption experiment using a bespoke adsorption column with recirculation. Adsorption percentage of MO using PANI-CS against time (V_{Total} = 200 ml, *Co* = 25 mg/L MO at pH = 4).

Time	% Adsorption PANI-CS
0	0.0
5	58.9
10	59.5
15	65.1
20	67.5
30	72.7
40	74.6
50	76.8
60	78.5
70	80.8
